# Shoulder Ultrasound in the Diagnosis of the Suprascapular Neuropathy in Athletes

**DOI:** 10.1515/med-2020-0022

**Published:** 2020-03-06

**Authors:** Barbara Igielska-Bela, Bogusław Baczkowski, Karol Flisikowski

**Affiliations:** 1 Department of Orthopedic, Specialist Hospital in Koscierzyna, ul.Piechowskiego 36, 83-400 Kościerzyna, Poland; 2 II Clinic of Orthopedy, Medical University of Gdansk, Gdansk Poland; 3 Department of Management and Economics, Gdansk University of Technology, Gdansk Poland

**Keywords:** Suprascapular neuropathy, Athletes, Ultrasound examination, Enthesopathy

## Abstract

**Purpose:**

Shoulder pain and weakness are common symptoms in athletes who play sports connected with overhead throwing. Suprascapular neuropathy may be one of the reason of such signs.The aim of the study was to find out if ultrasound examination of the shoulder in athletes reveals signs of suprascapular neuropathy.

**Methods:**

This was a cross-sectional study in which 67 professional volleyball, handball and rugby players of polish teams without shoulder disorders, which have played sport for 10 or more years, were included. An ultrasound examination of both shoulders was performed. Excluding criteria were recent shoulder and/or neck trauma and neurological disorders.

**Results:**

No ultrasound signs of suprascapular neuropathy were seen in any player. The only finding was enthesopathy of supraspinatus muscle and this finding was connected with athletes’ age, type of sport and with dominant hand. It had statistical significance with p-value respectively 0.01 for athletes’ age, 0.0208 for sport type and 0.03 for dominant hand.

**Conclusions:**

Ultrasonography should not be used as the screening examination of shoulders in athletes, but it can sometimes be an additional tool to help to diagnose shoulder disorders.

## 1 Introduction

Suprascapular neuropathy is a very rare neuropathy of upper limb nerves [2,3,5,7,12,16]. It was first described in 1936 [2,3,5,7,12]. It is one of the reasons of shoulder pain, especially due to sports play. Its frequency is about 1-2% [2,3,5,7,9]. Shoulder weakness, atrophy and diffuse aching or burning pain at the shoulder are signs of this neuropathy. These symptoms increase when the shoulder is in position of abduction above 90° and at night [1,4]. Unfortunately sometimes it can be asymptomatic [4].

Suprascapular nerve is a mixed motor and sensory nerve [3,4,16]. Its motor part is responsible for innervation of supraspinatus and infraspinatus muscles [4]. Its sensory branches go to the subacromial bursa, acromioclavicular and glenohumeral joints, and in some cases to the lateral side of the shoulder [4]. It starts from the superior trunk of the brachial plexus and goes down to suprascapular notch [4]. The nerve passes through this notch and then travels downward and outward to the spinoglenoid notch. It ends in the infraspinatus muscle. On its way it gives branches to innervated anatomical structures [4].

The mechanism of the injury of the suprascapular nerve has been described previously[1,4,8,15]. Suprascapular neuropathy is a common manifestation after trauma of peripheral branches of the brachial plexus, especially in athletes such as volleyball players or in sports which involve throwing [1,4,8,15].

The nerve can be damaged at suprascapular or spinoglenoid notch. The site of compression regulate the influence on the muscles [1,4,8,15].

Athletes who take part in repetitive activities experience neuropathy due to traction and micro trauma [1,4,8,15].

The mechanism is connected with increased pressure on the nerve, caused by tightening of the spinoglenoid ligament when the shoulder is in a position of overhead throwing [4].

Sometimes traction injury is secondary to rotator cuff tears.

The suprascapular nerve may also be compressed by a labral cyst. Such cysts develop from a labral rupture. Most cysts do not cause denervation of the muscle [4].

The diagnosis of suprascapular neuropathy include identification of symptoms, electromyography and sometimes MRI [1,4,7,16].

Ultrasound examination is known to be easy to perform, available, cheap, safe and does not expose the patient to radiation.

Ultrasound examination of shoulder is helpful in finding cysts or rotator cuff tears [7,10]. That is the reason why some investigators described also this method as a useful tool in diagnosing suprascapular neuropathy, especially in volleyball players [13,17]. There are some articles which suggest that ultrasound examination should be a screening examination in athletes [4,13].

The aim of the study was to perform ultrasound examination of the shoulder to check the signs of suprascapular neurpathy in high-performance volleyball, handball and rugby players.

## 2 Patients and methods

We started the investigation after ethical approval provided by the Bioethical Committee of Medical University. Every participant was asked to sign written informed consent.

The examinations have been performed in accordance with the ethical standards as laid down in the 1964 Declaration of Helsinki and its later amendments or comparable ethical standards.

The investigation was performed between April and July of 2017. Participants included were volleyball players, handball players and rugby players, who have played for about 10 years, both male and female. They had no episodes of shoulder trauma six months before the examination. They also had no neurological disorders.

The investigators were trained to perform ultrasound examination and had 5 years experience in musculoskeletal ultrasound.

We performed ultrasound examination using linear 5-12MHZ probe Sonoscape S8. All US scans were performed by the same doctor.

Participants were placed in sitting position and had their both shoulders examined. During ultrasound examination typical views of the shoulder were used, including supra- and infraspinatus muscles and the region of brachial plexus.

The data were collected using MS Excel 2010, and statistical analysis was made using Stata 14.2 and PQStat 1.6.4.

## 3 Results

We performed ultrasound examination in 67 professional athletes: 23 rugby players, 20 handball players and 24 volleyball players, with average age 27.4 (43% male and 57% female). The average period of sport play was about 10 years. In our investigation we did not find any case of suprascapular neuropathy.

**Figure 1:**
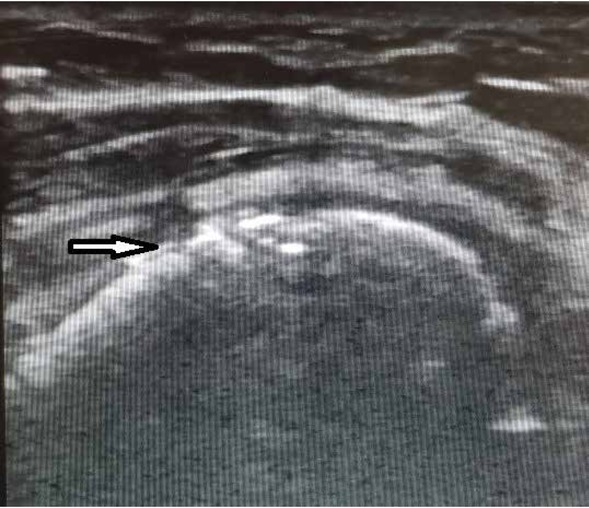
Irregular shape of humerał head (black arrow).

**Figure 2:**
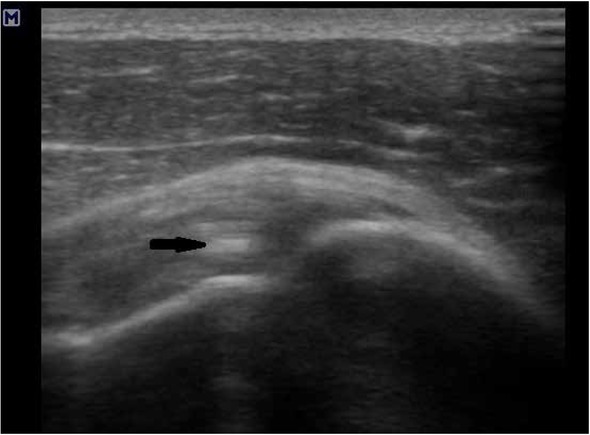
Calcyfications in supraspinatus tendon (black arrow).

The only finding was enthesopathy of supraspinatus muscle. It was imaged as irregular shape of humeral head and presence of calcifications in muscles’ tendons (Figure 1 and 2). This finding was statistically significant (using t-test in Stata 14.2) for age for both shoulders (Table 1).

**Table 1:** Demographic characteristics (USG R and L)

Characteristics	„normal” group R	„enthesopathy” group R	Values R	„normal” group L	„enthesopathy” group L	Values L
Age, mean (SD)	25.6 (8.53)	31.84 (9.05)	t=2.63, p=0.0107	26.66 (8.6)	35.16 (10.66)	t=2.265, p=0.026
Gender						
Male, n (%)	18 (37.5)	11 (57.89)	χ^2^=2.31	27 (44.26)	2 (33.33)	χ^2^=0.007^*^
Female, n (%)	30 (62.5)	8 (42.11)	p=0.1289	34 (55.74)	4 (66.67)	p=0.933^*^

* Yates’ correction for continuity

For right shoulder it was statistically depend on type of sport (Table 2). Because of little numbers of observations we show p-value using Fisher-Freeman-Halton test calculated in PQStat 1.6.4.

**Table 2:** Sport vs normal/enthesopathy (USG R)

Sport	“normal”, n (%)	“enthesopathy”, n (%)
Volleyball	18 (37.5)	6 (31.58)
Rugby	12 (25)	11 (57.89)
Handball	18 (37.5)	2 (10.53)

	χ^2^=7.74	p=0.0208^*^

* because of little numbers in sections p-value is shown using Fisher-Freeman-Halton test

Left shoulder enthesopathy of supraspinatus muscle was statistically significant for dominant hand (in this analysis we use Yates’ correction for continuity), (Table 3). There were no connections with athletes’ position in the team.

**Table 3:** Dominant hand vs normal/enthesopathy (USG L)

Dominant hand	“normal”, n (%)	“enthesopathy”, n (%)
P	59 (96.72)	4 (66.67)
L	2 (3.28)	2 (33.33)

	χ^2^=4.251^*^	p=0.039^*^

* Yates’ correction for continuity

This was a cross-sectional study the lack of a control group has to be mentioned.

## 4 Discussion

Although suprascapular neuropathy is a rare neuropathy, it is a common manifestation after injuries of the peripheral branches of the brachial plexus, especially in athletes such as volleyball players, or in sports requiring throwing with the upper limb set above the level of the shoulder, e.g. handball players or rugby players. The development of this neuropathy is favored by repetitive movements with arms raised above head and shoulder level.

Ultrasound examination is an effective tool in diagnosing disorders of musculoskeletal system.

The current study demonstrated no prevalence of suprascapular neuropathy in volleyball players, handball players and rugby players, as defined by ultrasound examination.

Nevertheless there are a lot of articles that show usefulness of sonography in diagnosing the suprascaular neuropathy.

The first case report was described in 1991 by Tokagishi et al [15]. They examined a 26 year old male with infraspinatus muscle atrophy and using ultrasound examination they found the ganglion cyst chich constricted the suprascapular nerve at the spinoglenoid notch [15].

Then, in 2000 Weiß et al. showed a case of a professional volleyball player who presented signs of suprascapular neuropathy [17]. The reason for this neuropathy was a cyst at suprascapular notch. They diagnosed this cyst during ultrasound examination of the shoulder. That was the reason why the authors suggested that because of common occurrence of such pathology in professional volleyball players, the ultrasound examination should be the screening method of the shoulders in athletes [17].

Despite this, the first report about suprascapular neuropathy in volleyball players was published in 1987 by Ferretti et al [6]. They examined ninety-six top-level volleyball players from eight teams using electromyography and isokinetic dynamometry. They found twelve players had a symptomatic isolated paralysis of the infraspinatus muscle [6].

In 2000 Witvrouw et al. presented a study with 16 professional volleyball players [18]. They performed an electromyographic investigation, a clinical shoulder examination, checked shoulder range of motion and an isokinetic concentric peak torque shoulder internal/external rotation strength test [18]. They suggested that there is an association between increased range of motion of the shoulder joint and the presence of isolated paralysis of the infraspinatus muscle in volleyball players [18].

The question about frequency of suprascapular neuropathy in high performance volleyball players was described in 2014 by Pieber et al [14]. In their study they did not find a lesion of suprascapular nerve [14]. A similar study was made by Lajtai et al [11]. But in their investigation they recommended further EMG-based studies to confirm the hypothesis that the reason of infraspinatus atrophy in volleyball players is a stretching neuropathy of the suprascapular nerve, caused by repetitive hitting activity [11].

There is lack of articles about the ultrasound examination in handball or rugby players.

According to our results we found no sonographic signs of suprascapular neuropathy. The only finding was enthesopathy of supraspinatus muscle. The enthesopathy is also called calcifying inflammatory of the muscles’ tendon (tendinitis) . This disorder is very common after overloading the shoulder.

An interesting observation resulting from the conducted research is the dependence of the occurring enthesopathy of the supraspinatus muscle tendon on the age, type of sport and the dominant hand. This is due to degenerative changes in the muscle after prolonged maintenance of the upper limb above head level and overloading resulting from playing sports. The relationship with age is due to the body’s ageing processes. The relationship with the type of sport probably results from overloading due to the characteristic movements for the type of sport. While the dependence on the dominant hand results from more frequent use of this hand. In the examined athletes, changes in ultrasound were visible despite the absence of shoulder pain or previous shoulder injuries. This may suggest that ultrasound symptoms are ahead of the clinical symptoms and only some external factor “triggers” their appearance.

The study group was numerous, so this is not a limitation of the study. Additionally, in this study athletes had practised their sports for a minimum of 10 years. They were professional athletes, not practicing recreational sports. The group was representative to perform the study. The only noticeable limitation could be the lack of nerve conduction studies. The results of this examination could be additionally correlated with the results of the ultrasound examination. It has been shown that there is no need for screening shoulder ultrasound on volleyball players and other athletes, such as handballers and rugby players. However, this test is effective when suspecting shoulder disorders and physicians should remember about this simple diagnostic method.

## 5 Conclusions

We conclude that although volleyball, handball or rugby players may present atrophy of supra- and infraspinatus muscles with or without pain in their shoulder, no ultrasound signs of suprascapular neuropathy was found in the study population. We think that our population group was large enough and this is the reason why we think that there is no need to use ultrasound as a screening examination of shoulder in athletes. We also believe that ultrasound examination can help to establish when there are signs of this disorder.
